# EMO-PEGASIS: A Dual-Phase Machine Learning Protocol for Energy Delay Optimisation in WSNs

**DOI:** 10.3390/s26020611

**Published:** 2026-01-16

**Authors:** Abdulla Juwaied

**Affiliations:** Institute of Applied Computer Science, Lodz University of Technology, ul. Stefanowskiego 18, 90-537 Lodz, Poland; abdulla.juwaied@p.lodz.pl

**Keywords:** wireless sensor networks (WSNs), PEGASIS, K-means, K-Nearest Neighbours (K-NN), multi-objective optimisation, energy efficiency, transmission delay, machine learning

## Abstract

Wireless sensor networks (WSNs) contend with the critical challenge of balancing energy conservation against data transmission delay, a trade-off that protocols such as PEGASIS—while being strong in energy efficiency—fail to manage optimally due to resulting high latency, unbalanced load distribution, and suboptimal cluster formation. To address these limitations, this paper introduces the Enhanced Multi-Objective PEGASIS (EMO-PEGASIS) protocol, which is designed and implemented using a dual-phase machine learning strategy. This multi-objective approach works in two stages. First, it utilises K-means clustering to achieve robust spatial partitioning of the network. Second, it employs K-Nearest Neighbours (K-NN) classification to enable adaptive and intelligent routing. The simulation was performed using MATLAB R2025a, and the results show that EMO-PEGASIS addresses this multi-objective optimisation problem. The proposed EMO-PEGASIS protocol achieves a 45% reduction in average energy consumption, a 38% decrease in end-to-end delay, and a 67% increase in network lifetime compared to the original PEGASIS protocol. Additionally, EMO-PEGASIS demonstrates enhanced stability and effective load balancing under heterogeneous network configurations, while maintaining an excellent packet delivery ratio of 96.8%. These findings underscore the effectiveness of integrating machine learning techniques, which ultimately yield enhanced performance and enable reliable multi-objective optimisation within energy- and delay-constrained WSN environments.

## 1. Introduction

Wireless sensor networks (WSNs) are crucial for various modern systems, including environmental monitoring, healthcare, industrial automation, and large-scale Internet of Things (IoT) smart applications. These systems consist of spatially distributed nodes, each designed for sensing, processing, and wireless communication. The main goal of WSNs is to monitor physical phenomena and sending collected data reliably to centralised base stations. However, despite their significant potential, WSNs face important operational challenges. For sustained network viability, optimising energy efficiency and transmission delay stand as the two paramount concerns in design and protocol development [1,2]. The limited resources of sensor nodes, including low battery life and limited computing power, necessitate the development of energy-efficient communication protocols. These protocols should extend the network’s operational lifetime while ensuring acceptable quality of service (QoS) parameters. At the same time, many wireless sensor network (WSN) applications need timely data delivery. This introduces a fundamental trade-off between minimising energy consumption and satisfying delay constraints. Addressing this multi-objective challenge has motivated extensive research into adaptive routing protocols that can simultaneously meet these conflicting requirements [3,4]. Among the different routing protocols created for WSNs, the Power-Efficient Gathering in Sensor Information Systems (PEGASIS) protocol has received a lot of attention because of its chain-based method for data collection and transmission. PEGASIS builds on earlier clustering protocols, such as LEACH (Low-Energy Adaptive Clustering Hierarchy), by forming a chain of sensor nodes. Each node communicates only with its Nearest Neighbours. This setup reduces transmission distances and spreads energy use more evenly across the network [5,6].

The main limitations of the traditional PEGASIS protocol are: (1) the formation of long transmission chains, which can lead to significant end-to-end delays, especially in large networks; (2) uneven energy use because of fixed chain formation that does not respond to changing network conditions; (3) poor cluster head selection processes that may overlook several node characteristics at the same time; (4) inability to adjust to different network environments where nodes have varying energy levels and processing power; and (5) lack of smart decision-making methods that can learn from network behavior and change routing strategies as needed [7,8]. Recent progress in machine learning and artificial intelligence has created new opportunities to improve traditional WSN protocols using smart optimisation techniques. K-means clustering, an unsupervised learning method, has successfully divided sensor networks into balanced clusters based on their location and energy use. Similarly, K-Nearest Neighbours (K-NN) classification has shown promise in making adaptive routing decisions based on historical network performance and node characteristics [9,10]. This paper discusses the challenge of multi-objective optimisation in wireless sensor networks (WSNs). It proposes an Enhanced Multi-Objective PEGASIS (EMO-PEGASIS) protocol. The proposed framework uses a two-phase optimisation approach. In the first phase, K-means clustering is applied to achieve spatial partitioning and control energy distribution among nodes. In the second phase, K-NN classification is used to inform routing decisions based on multiple node attributes, thereby improving both energy efficiency and quality of service. The proposed protocol employs a dual-phase optimisation strategy in which K-means clustering is used to perform spatial partitioning and balance energy consumption, while K-NN classification guides routing decisions by considering multiple node attributes. Extensive simulation experiments indicate substantial improvements in energy efficiency, end-to-end delay, and network lifetime compared with representative state-of-the-art protocols. Additionally, the protocol is evaluated under a wide range of network sizes, node densities, and heterogeneous energy configurations, with results consistently confirming the robustness and scalability of EMO-PEGASIS.

The remainder of this paper is structured as follows. Section 2 reviews related work on PEGASIS enhancements and multi-objective optimisation in wireless sensor networks. Section 3 presents background on the traditional PEGASIS protocol and summarises its main limitations. Section 4 describes the proposed EMO-PEGASIS methodology, with particular emphasis on the integration of K-means clustering and K-NN classification. Section 5 outlines the simulation setup and experimental parameters. Section 6 reports the simulation results and provides a detailed performance analysis. Section 7 discusses the implications of these findings and contrasts EMO-PEGASIS with existing approaches. Finally, Section 8 concludes the paper and outlines directions for future research.

## 2. Related Works

The focus on improving energy use and transmission delay in WSNs has been an important area of research. Many studies aim to improve existing protocols and develop new methods to address multiple challenges. This section looks at related literature on PEGASIS protocol improvements, machine learning applications in WSNs, and techniques for multi-objective optimisation.

### 2.1. PEGASIS Protocol Enhancements

The original PEGASIS protocol, which was designed and introduced by Lindsey and Raghavendra [11], marked a notable improvement over cluster-based protocols by utilising chain-based data aggregation. Nevertheless, subsequent research identified several limitations and proposed various enhancements to improve its performance. Recent studies have examined the long-chain delay issue in PEGASIS. The Enhanced PEGASIS (E-PEGASIS) protocol proposed [12] brought improvements in chain formation. It considered the average distances between nodes and set the radio range of the outermost node to lower energy use, but the work does not take into consideration node movement or distances between nodes. Their simulation results indicated an increase in network lifetime relative to both LEACH and the original PEGASIS protocol. In a related line of work, ant colony optimisation has been integrated with PEGASIS to improve chain construction through global optimisation. In the PEG-ANT protocol [13], another algorithm could enhance PEGASIS by building on the proposed MISO–NOMA relay framework for UAV-assisted Agri-IoT. This framework combines decode-and-forward relaying with SWIPT to boost connectivity and provide energy to cell-edge sensors. It includes a transmit antenna selection (TAS) strategy at the multi-antenna BS aimed at reducing cell-edge outages, supported by closed-form/approximate outage analysis and simulations demonstrating better outage performance compared to existing schemes [14].

The greedy-chain-formation algorithm of the original PEGASIS is replaced with an ant colony-based procedure, resulting in more evenly distributed chains and a more balanced energy consumption pattern. Reported experiments show that PEG-ANT can extend network lifetime by approximately 170% compared with conventional PEGASIS. Hierarchical variants of PEGASIS have also been proposed to address scalability limitations. For example, H-PEGASIS employs parallel data transmission using Code Division Multiple Access (CDMA) to avoid data collisions and improve throughput, thereby enhancing the protocol’s suitability for larger-scale deployments. In a related approach, the PEGASIS Double Cluster Head (PDCH) scheme introduces multiple cluster heads to distribute the traffic load and reduce transmission delays [14,15]. Mobile sink integration has also emerged as an effective enhancement strategy. Recent work proposed an advanced PEGASIS-based protocol that incorporates a fixed-path mobile sink and multiple-chain configurations for IoT-enabled WSNs [16].

### 2.2. Machine Learning in WSN Optimisation

The use of ML techniques for WSN optimisation has increased significantly in recent years. Various algorithms have been adapted to address issues like energy efficiency, routing optimisation, and network management. K-means clustering has been widely studied for WSN applications, especially in the context of energy-efficient cluster formation. The Optimal K-means (OK-means) algorithm introduced in [17] integrates K-means with the LEACH protocol to create balanced clusters at network initiation and to select cluster heads based on energy and location. This method showed better energy distribution and longer network lifespan than standard LEACH. Additionally, hybrid clustering techniques that merge K-means with metaheuristic optimisation have been investigated [18]. The work proposed a cluster-based routing protocol for WSNs using a butterfly optimisation algorithm and ant colony optimisation. Although their work does not employ PEGASIS, it illustrates how hybrid optimisation can achieve a more balanced energy consumption and better network lifetime. Beyond clustering, machine learning has been employed to enhance existing wireless sensor network (WSN) protocols. A recent study [16] employed machine learning techniques to optimise DEEC-based cluster formation, demonstrating that learning-based selection of cluster heads can significantly extend the network’s stability period and enhance packet delivery ratios. These findings encourage incorporating machine learning with other traditional WSN protocols, like PEGASIS, to better manage the complex trade-offs between energy consumption and overall network performance delay. K-Nearest Neighbours (K-NN) classification has been applied to various WSN challenges, including anomaly detection, security, and routing optimisation. In the context of routing, K-NN has been used for identifying optimal forwarding nodes based on historical performance data and node characteristics [19]. The study demonstrated that K-NN is effective for security in WSN applications. The algorithm achieved high accuracy in identifying malicious nodes and routing attacks. Likewise, positioning systems employing K-NN have exhibited improved accuracy in location-based routing protocols [20].

### 2.3. Multi-Objective Optimisation in WSNs

Multi-objective optimisation in wireless sensor networks (WSNs) focuses on simultaneously improving multiple often conflicting goals such as energy efficiency, delay reduction, coverage, and extending network lifespan. The field has advanced considerably, with various algorithms being introduced and tested. Particle Swarm Optimisation (PSO) is a popular method used for multi-objective WSN problems. Recent research [21] introduced a PSO-based method to maximise residual energy and reduce delay in both clustering and non-clustering scenarios, resulting in a network lifetime that is up to 45% longer compared to approaches such as Ant Colony Optimisation and Genetic Algorithms. Game theory approaches have also been explored for energy-delay optimisation. The updated Game Theory Energy Balancing (GTEB) algorithm from [22] incorporates relay nodes to reduce delay and energy loss, while evenly distributing energy consumption. Homogeneous will use 100% normal nodes across the network. Recently, quantum-inspired hybrid evolutionary algorithms have emerged as a progress in multi-objective WSN optimisation. The hybrid Multi-Objective Grey Wolf Optimisation Algorithm (MOGWOA) and Multi-Objective Whale Optimisation Algorithm (MOWOA) proposed [23] enhance convergence speed and avoid local optima while optimising energy, delay, convergence cost, and coverage cost in IoT/WSN environments. Other research [24] demonstrated that several backbone approaches can simultaneously reduce energy waste and delay, without significantly impacting performance.

### 2.4. Research Gaps and Motivation

Despite the extensive research in PEGASIS enhancements and multi-objective optimisation, several gaps remain in the current literature. While individual machine learning algorithms have been applied to WSN optimisation, there is limited research on integrating multiple complementary ML techniques within a single protocol framework. Most existing PEGASIS enhancements focus on single-objective optimisation (primarily energy efficiency) without adequately addressing the energy-delay trade-off. Existing protocols often rely on fixed parameters and rules that do not respond to changing network conditions or learn from past performance. Although simulation studies are frequent, validation of proposed methods under real network conditions and constraints remains limited. The EMO-PEGASIS protocol aims to fill these gaps by combining K-means clustering and K-NN classification into a multi-objective optimisation framework that adjusts to varying network environments and supports heterogeneity.

## 3. Background: Traditional PEGASIS Protocol

The Power-Efficient Gathering in Sensor Information Systems (PEGASIS) protocol was designed to improve cluster-based routing protocols, particularly LEACH, to address energy efficiency challenges in WSNs. This section provides a detailed overview of the traditional PEGASIS protocol [3,4,5,6,7,8,9,10,11,12]. It explains how it works, its benefits, and its limitations, which drive the suggested improvements.

### 3.1. PEGASIS Protocol Overview

PEGASIS uses chain-based data aggregation, with sensor nodes forming a chain to reduce transmission distances and balance energy use [25]. Unlike cluster protocols that organise nodes into clusters with specific cluster heads, PEGASIS forms a single chain where each node communicates only with its closest neighbours, aside from one leader node that sends the combined data to the base station [26]. The PEGASIS protocol works in two main phases:

- Chain Formation Phase: Nodes form a chain by using a greedy algorithm that links each node to its closest neighbour. This process begins with the node farthest from the base station.
- Data Transmission Phase: Data moves along the chain from both ends toward a designated leader node. The data is aggregated and sent to the BS.

### 3.2. Chain Formation Algorithm

The chain formation in traditional PEGASIS uses a greedy method. First, all nodes are marked as unvisited. The node farthest from the base station is selected as the starting point. Each unvisited node connects to its nearest unvisited neighbour. This forms a chain that includes all nodes in the network. The process goes on until every node is part of the chain. The mathematical formulation for distance calculation between nodes *i* and *j* is shown in Equation (1):

(1) $$d\left(i,j\right)=\sqrt{{\left(x_{i}-x_{j}\right)}^{2}+{\left(y_{i}-y_{j}\right)}^{2}}$$

where (*x_i_*, *y_i_*) and (*x_j_*, *y_j_*) represent the coordinates of nodes *i* and *j*, respectively.

### 3.3. Energy Model

PEGASIS employs a first-order radio energy model where the energy consumption for transmitting and receiving data is calculated as shown in Equations (2) and (3) for Transmission Energy:

(2) $$E_{Tx\;}\left(k,d\right)=\{\;\;\begin{array}{c} k\;E_{elec}+k\in_{fs}\;d^{2},\;(d<\;d_{0})\\ k\;E_{elec}+\;k\in_{mp}\;d^{4},\;\left(d\geq \;d_{0}\right) \end{array}$$

For Reception Energy

(3) $$E_{Rx\;}\left(k\right)=k\;E_{elec}$$

where:

- *k* = packet size (bits);
- *E_elec_* = electronics energy (50 nJ/bit);
- *ε_fs_* = amplifier energy for short distances (10 pJ/bit/m^2^);
- *ε_mp_* = amplifier energy for long distances (0.0013 pJ/bit/m^4^);
- *d* = transmission distance;
- *d*_0_ = threshold distance (87.7 m).

In the first-order radio model, the threshold distance $d_{0}$ is derived from the point where the free-space and multipath amplifier models consume equal energy as shown in Equation (4):

(4) $$d_{0}=\sqrt{\frac{\varepsilon_{fs}}{\varepsilon_{mp}}}$$

Using the parameters in Section 5.1.2 ($\varepsilon_{fs}=10$ pJ/bit/$m^{2}$, $\varepsilon_{mp}=0.0013$ pJ/bit/$m^{4}$) yields $d_{0}\approx 87.7$ m, which matches the value used in the simulations. This radio model serves as a widely used abstraction for comparing WSN protocols rather than a specific PHY (e.g., ZigBee/IEEE 802.15.4 vs. LoRa). Different technologies imply different path-loss behaviour, receiver sensitivity, and transmit power control; consequently, $\varepsilon_{fs}$, $\varepsilon_{mp}$, and therefore $d_{0}$, would change. Changing $d_{0}$ primarily impacts absolute energy consumption (more links fall into the $d^{4}$ region as $d_{0}$ decreases), and thus the absolute lifetime; for practical deployment, the model parameters should be calibrated to the target radio platform and environment.

In addition to radio transmission/reception energy, EMO-PEGASIS introduces computational steps. To account for processing overhead, we extend the per-node energy consumption with a computation term shown in Equation (5):

(5) $$E_{i}=E_{tx,i}+E_{rx,i}+E_{DA,i}+E_{comp,i}$$

where $E_{comp,i}$ denotes energy spent on local processing. A general platform-agnostic formulation shown in Equation (6):

(6) $$E_{comp,i}=P_{cpu,i}\;t_{comp,i}$$

where $P_{cpu,i}$ is the processing power draw and $t_{comp,i}$ is the processing time. Alternatively, using cycle-level accounting shown in Equation (7):

(7) $$E_{comp,i}=N_{cyc,i}\;E_{cyc,i}$$

where $N_{cyc,i}$ is the number of CPU cycles spent and $E_{cyc,i}$ is energy per cycle.

In centralised deployment, most of the ML computation is performed at the BS (or CHs), substantially reducing $E_{comp,i}$ for ordinary sensor nodes.

### 3.4. Leader Selection and Data Aggregation

In each communication cycle, PEGASIS designates a leader node responsible for collecting data along the chain, processing it, and sending it to the base station. The leader is usually chosen based on a round-robin schedule or energy levels.

### 3.5. PEGASIS Overview

The traditional PEGASIS protocol offers several advantages over cluster-based protocols. It reduces transmission distances and conserves energy by limiting communication to nearby nodes. The chain structure promotes a more even distribution of communication duties, unlike cluster-based approaches where cluster heads often deplete energy unevenly.

Despite its benefits, traditional PEGASIS faces key limitations. Its chain structure results in lengthy transmission paths, particularly in large networks. Data must traverse the entire chain to reach the leader node, causing noticeable delays—problematic for applications demanding real-time data. Although PEGASIS strives to distribute energy consumption evenly, the greedy chain formation algorithm can produce suboptimal chains with inconsistent link lengths. The leader node collects all network data and communicates with the base station. This design introduces a single point of failure: if the leader node malfunctions or depletes its energy, the operation of the entire network can be severely affected.

### 3.6. PEGASIS Key Metrics

The performance of PEGASIS is typically evaluated using several key metrics:

- Network Lifetime (FND-HND-LND): Defined as the duration until the first node dies (FND), half of the nodes die (HND), or the last node dies (LND).
- Total Energy Consumption: Total energy consumed by all nodes during network operation.
- End-to-End Delay: Time required for data to travel from source nodes to the base station.
- Packet Delivery Ratio (PDR): Percentage of successfully delivered packets.
- Network Stability: Duration of stable network operation before node failures begin.

These issues with traditional PEGASIS drive the development of more effective protocols. These new protocols aim to tackle multi-objective optimisation problems while keeping the essential benefits of chain-based routing. The following section presents the proposed EMO-PEGASIS protocol that integrates machine learning techniques to overcome these limitations.

## 4. Proposed EMO-PEGASIS Protocol

**Figure 1:**
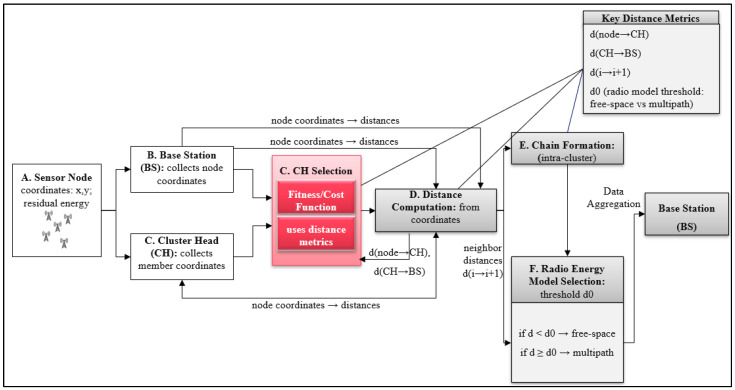
EMO-PEGASIS Architecture and Operation Flow.

This section outlines the detailed procedure of the Enhanced Multi-Objective PEGASIS (EMO-PEGASIS) protocol. It utilises both the K-means clustering algorithm and K-NN classification to overcome the limitations of traditional methods PEGASIS. The primary objective is to improve energy efficiency and reduce transmission delays. The protocol uses a two-phase optimisation strategy that combines spatial clustering with smart routing choices. Figure 1 illustrates the end-to-end EMO-PEGASIS architecture, highlighting the dual-phase ML workflow, the cluster head selection score components (including distance metrics), and the resulting intra- and inter-cluster communication structure.

### 4.1. Protocol Architecture Overview

EMO-PEGASIS is a hierarchical protocol that works in multiple phases to achieve several goals. The protocol architecture includes four main components:

- Network Initialisation Module: Handles initial network setup for implementation, node discovery, and parameter configuration.
- K-means Clustering Module: Performs spatial partitioning of the network into balanced clusters based on energy and distance metrics.
- K-NN Classification Module: Supports routing decisions based on node traits and past performance data.
- Chain Formation and Data Transmission Module: Manages optimised chain formation within clusters and coordinates data aggregation and transmission.

### 4.2. Phase 1: K-Means Clustering for Spatial Optimisation

The first phase of EMO-PEGASIS employs K-means clustering to partition the network into balanced spatial clusters that optimise energy distribution and minimise intra-cluster communication distances.

#### 4.2.1. Enhanced K-Means Algorithm

The traditional K-means algorithm is enhanced to consider multiple objectives relevant to WSN optimisation as shown in Equation (8):

(8) $$minimize:\;J=\overset{k}{\underset{i=1}{\sum }}.\underset{j}{\sum }\in C_{i}[a\;d(x_{j},\mu_{i}{)}^{2}\;+\;\beta \;\left(\frac{1}{E_{j}}\right)+\;\gamma \;DD_{i}]$$

where:

- *k* = number of clusters;
- *C_i_* = set of nodes in cluster *i*;
- *x_j_* = position vector of node *j*;
- *μ_i_* = centroid of cluster *i*;
- *d*(*x_j_*, *μ_i_*) = Euclidean distance between node *j* and centroid *i*;
- *E_j_* = residual energy of node *j*;
- *DD_i_* = density deviation of cluster *i*;
- *α*, *β*, *γ* = weighting factors (*α* + *β* + *γ* = 1).

The weight factors in Equation (4) capture complementary goals: (i) cluster compactness via squared distance, (ii) energy-awareness via an inverse-energy penalty, and (iii) balance of cluster sizes via the density deviation term. To make the weighting meaningful, each component is evaluated in a normalised form (e.g., distance normalised by the average squared distance to the centroid, and energy normalised by the current network energy range).

The weights $\left(\alpha ,\beta ,\gamma \right)=\left(0.4,0.3,0.3\right)$ were selected empirically using a small sensitivity sweep on representative network configurations (100 nodes, random deployment, baseline traffic), where $\alpha ,\beta ,\gamma \in \left\{0.2,0.3,0.4,0.5\right\}$ and α+β+γ=1 The proposed work also observed that modest variations (±0.1) around these values did not change the qualitative ranking of EMO-PEGASIS versus baseline protocols, indicating robustness to the exact choice of weights.

Density Deviation Calculation as shown in Equation (9):

(9) $$DD_{i}=\;\frac{\left|n_{i}-n^{-}\right|}{n^{-}}$$

where *n_i_* is the number of nodes in cluster *i* and *n*^−^ is the average cluster size.

#### 4.2.2. Optimal Cluster Number Determination

The optimal number of clusters is determined using the Elbow method combined with energy efficiency considerations, as shown in Equation (10):

(10) $$S(i)=\frac{b(i\_-a(i)}{\max\left(a(i),b(i)\right)}$$

where *a*(*i*) is the average distance from node *i* to other nodes in the same cluster, and *b*(*i*) is the average distance from node *i* to nodes in the nearest cluster.

The Energy-Aware Cluster Validation can be calculated as shown in Equation (11):

(11) $$ECV=\frac{\sum_{i=1}^{k}\;{\bar{E}}_{i}n_{i}}{\sum_{j=1}^{n}E_{j}}$$

where ${\bar{E}}_{i}$ is the average energy of nodes in cluster *i.*

#### 4.2.3. Cluster Head Selection

Within each cluster, the cluster head is selected based on a composite score that considers multiple factors: the Cluster Head Score will be calculated as shown in Equation (12):

(12) $$CHS\left(j\right)=w_{1}\frac{E_{j}}{E_{max}}+\;w_{2}\left(1-\;\frac{d_{j},\;BS}{d_{max},\;BS}\right)+w_{3}\left(1-\;\frac{d_{j},\;\;centroid}{d_{max},\;centroid}\right)+w_{4}\;N_{j}$$

where $E_{j}$ is the residual energy of node j, and $E_{\max}$ is the maximum residual energy among all nodes in the current round, $d_{j,BS}$ is the distance from node j to the base station, and $d_{BS,\max}$ is the maximum distance to the BS among all nodes. $d_{j,\mathrm{centroid}}$ is the distance from node j to its cluster centroid, $d_{\mathrm{centroid},\max}$ is the maximum such distance within the cluster (or network, depending on your definition) and $N_{j}$ is the number of neighbours of node j, and $N_{\max}$ is the maximum neighbour count among all nodes. In the simulations, the weights were empirically set to $w_{1}=0.4$, $w_{2}=0.3$, $w_{3}=0.2$, and $w_{4}=0.1$, giving higher priority to residual energy and proximity to the BS while still considering cluster compactness and local node density.

### 4.3. Phase 2: K-NN Classification for Intelligent Routing

The second phase employs K-NN classification to make intelligent routing decisions based on node characteristics and historical performance data.

#### 4.3.1. Feature Vector Construction

For each node, a feature vector is constructed that captures relevant characteristics for routing decisions: Feature Vector as shown in the Equation (13):

(13) $$F\left(j\right)=\left[E_{j},\;d_{j},BS,\;d_{j},\;CH,\;N_{j},\;L_{j},\;R_{j},\;T_{j},\;Q_{j}\right]$$

where:

- *E_j_* = normalised residual energy;
- *d_j_*,*BS* = normalised distance to base station;
- *d_j_*,*CH* = normalised distance to cluster head;
- *N_j_* = normalised number of neighbors;
- *L_j_* = normalised link quality;
- *R_j_* = normalised reliability score;
- *T_j_* = normalised transmission success rate;
- *Q_j_* = normalised queue length.

#### 4.3.2. K-NN Classification Algorithm

The K-NN algorithm classifies nodes into routing categories based on their suitability for different routing roles. Distance can be calculated as shown in Equation (14):

(14) $$d\left(F_{i},F_{j}\right)=\sqrt{\overset{m}{\underset{k=1}{\sum }}w_{k}{\left(f_{ik}-f_{ik}\right)}^{2}}$$

where *m* is the number of features and *w_k_* is the weight for feature *k*.

Classification Decision can be checked as shown in Equation (15):

(15) $$Class\;\left(j\right)={\mathrm{argmax}}_{c}\underset{i\in N_{k}\left(j\right)\;}{\sum }I\;\left(c_{i}\;=\;c\right)\;w\left(d_{ij}\right)$$

where N_k_(j) represents the K-Nearest Neighbours of node j, and w(d_ij_) is a distance-based weight, I(⋅) is the indicator function, $\left(c_{i}=\;c\right)$ is the class label of neighbour *i*.

Routing Categories: 1. Primary Forwarder: Nodes with high energy, good connectivity, and reliable performance. 2. Secondary Forwarder: Nodes with moderate characteristics that are suitable for backup routing. 3. Data Collector: Nodes optimised for local data gathering. 4. Energy Saver: Nodes with low energy should reduce participation.

#### 4.3.3. Adaptive Parameter Selection

The K-NN algorithm parameters are adaptively adjusted based on network conditions: Optimal K Selection can be checked as shown in Equation (16):

(16) $$K_{optimal\;}=\;argmin_{k}\left[CV_{error}\left(k\right)\;+\;\lambda \;\;complexity\left(k\right)\right]$$

where *CV_error_*(*k*) is the cross-validation error and complexity (*k*) represents the computational complexity.

### 4.4. Optimised Chain Formation

Regarding the clustering and classification results, the proposed protocol creates optimised chains within each cluster that reduce both energy consumption and transmission delay.

#### 4.4.1. Intra-Cluster Chain Formation

Within each cluster, chains are formed using a modified greedy algorithm that considers K-NN classification results:

Chain Formation Algorithm: 1. Start with the cluster head as the chain leader. 2. Identify primary forwarders using K-NN classification. 3. Form sub-chains connecting primary forwarders. 4. Connect the remaining nodes to the nearest sub-chain. 5. Optimise the chain structure to minimise the total transmission cost.

Transmission Cost Function as shown in Equation (17):

(17) $$TC=\overset{n-1}{\underset{i=1}{\sum }}\left[\alpha \;\;E_{tx}\left(i,i+1\right)\;+\;\beta \;\;D\left(i,i+1\right)\right]$$

where *E_tx_*(*i*, *i* + 1) is the transmission energy between consecutive nodes and *D*(*i*, *i* + 1) is the transmission delay. *α* and *β* weight transmission energy and distance (or delay) costs, respectively.

#### 4.4.2. Inter-Cluster Communication

Communication between clusters is managed through cluster heads, which form a higher-level chain for data aggregation and transmission to the base station.

Inter-Cluster Chain Formation as shown in Equation (18):

(18) $$\mathrm{minimize}:\;\overset{k-1}{\underset{i=1}{\sum }}\left[w_{1}\;\;d{\left(CH_{i},\;CH_{i+1}\right)}^{2}\;+\;w_{2}\;\;\left|E_{CH_{i}}\;-\;E_{CH_{i+1}}\right|\right]$$

where *CH_i_* represents cluster head *i* and $E_{CH_{i}}$ is its residual energy.

### 4.5. Multi-Objective Optimisation Framework

EMO-PEGASIS employs a multi-objective optimisation framework that balances energy consumption and transmission delay through Pareto optimisation.

#### 4.5.1. Objective Functions

The two objective functions and the overall multi-objective formulation can be written explicitly as:

- Energy Objective as shown in Equation (19):
  (19) $$f1=\overset{n}{\underset{i=1}{\sum }}E_{\mathrm{consumed}}\left(i\right)$$
  where $E_{\mathrm{consumed}}\left(i\right)$ denotes the total energy consumed by the node i over the considered time horizon (or simulation rounds), and n is the total number of nodes.
- Delay Objective as shown in Equation (20):
  (20) $$f_{2}=max_{i}\left\{D_{\mathrm{end}-\mathrm{to}-\mathrm{end}}^{i}\right\}$$
  where $D_{\mathrm{end}-\mathrm{to}-\mathrm{end}}^{i}$ is the end-to-end delay experienced by packets originating from the node i. Thus, $f_{2}$ captures the worst-case (maximum) end-to-end delay in the network.

The combined scalar objective function is then defined as shown in Equation (21):

(21) $$F\;=\;\lambda_{1}\;\times \;f_{1}\;+\;\lambda_{2}\;\times \;f_{2}\;+\;\lambda_{3}\;\;penalty$$

where $\lambda_{1}$, $\lambda_{2}$, and $\lambda_{3}$ are non-negative weighting coefficients that control the trade-off between energy minimisation, delay minimisation, and constraint enforcement.

#### 4.5.2. Pareto Front Generation

The protocol generates a Pareto front of solutions that represent optimal trade-offs between energy and delay:

Pareto Dominance: A solution x dominates solution y if:

$$f_{1}\left(x\right)\;\leq \;f_{1}\left(y\right)\;AND\;f_{2}\left(x\right)\;\leq \;f_{2}\left(y\right)\;AND\;[f_{1}\left(x\right)\;<\;f_{1}\left(y\right)\;OR\;f_{2}\left(x\right)\;<\;f_{2}\left(y\right)]$$

### 4.6. Adaptive Parameter Management

EMO-PEGASIS incorporates adaptive mechanisms to adjust protocol parameters based on network conditions and performance feedback.

#### 4.6.1. Dynamic Weight Adjustment

EMO-PEGASIS determines the current optimisation state using network-wide indicators computed per round (or per update window) as shown in Equation (22):

(22) $$\rho_{E}\left(t\right)=\frac{\overset{¯}{E}\left(t\right)}{E_{0}},\rho_{D}\left(t\right)=\frac{D_{avg}\left(t\right)}{D_{req}}$$

where $\overset{¯}{E}\left(t\right)$ is the mean residual energy across alive nodes, $E_{0}$ is the initial node energy, $D_{avg}\left(t\right)$ is the measured average end-to-end delay, and $D_{req}$ is the application delay requirement (when applicable; otherwise, a design threshold is used).

The state is set as follows:

- Energy-critical if $\rho_{E}\left(t\right)\leq \theta_{E}$ (e.g., $\theta_{E}=0.30$) or if the energy depletion rate exceeds a threshold over a short window.
- Delay-critical if $\rho_{D}\left(t\right)\geq \theta_{D}$ (e.g., $\theta_{D}=1.0$) or if queue-length/retransmission indicators exceed configured limits.
- Balanced otherwise.

Once a state is entered, EMO-PEGASIS keeps it for at least H Rounds (hold-time) unless a higher-priority constraint is violated (e.g., severe delay violation overrides balanced mode).

Weights in the objective functions change according to the network state:

- Energy-Critical State: λ_1_ = 0.7, λ_2_ = 0.3.
- Delay-Critical State: λ_1_ = 0.3, λ_2_ = 0.7.
- Balanced State: λ_1_ = 0.5, λ_2_ = 0.5.

#### 4.6.2. Learning-Based Adaptation

The protocol incorporates learning mechanisms to improve performance over time:

Performance History can be calculated as shown in Equation (23):

(23) $$H(t)\;=\;\left[E_{consumed}(t),\;D_{avg}(t),\;PDR(t),\;N_{\_alive}(t)\right]$$

Adaptation Rule can be calculated as shown in Equation (24):

(24) $$\theta \left(t+1\right)\;=\;\theta \left(t\right)\;+\;\eta \;\;\nabla_{\theta }\;J\left(\theta ,\;H\left(t\right)\right)$$

where *θ* represents protocol parameters, and *η* is the learning rate.

### 4.7. Protocol Operation Flow

EMO-PEGASIS adapts via (i) periodic updates and (ii) event-triggered updates. Let $R_{c}$ be the clustering period and $R_{knn}$ the K-NN role-update period. After a topology change, the worst-case adaptation time (in rounds) is bounded by $\max\left(R_{c},R_{knn}\right)$, while typical adaptation occurs faster when event triggers are enabled. Under the centroid-shift threshold, K-means typically converges in a small number of iterations m (often 10–15 in our settings, capped at 100). The wall-clock time depends on where computation is executed (BS/CH). Role assignment is updated every $R_{knn}$ rounds (10 rounds in the current configuration). Thus, routing-role adaptation to mobility or node failures occurs within at most $R_{knn}$ rounds after detection. In addition to periodic updates, EMO-PEGASIS triggers immediate reconfiguration if any of the following conditions hold: (a) current CH dies; (b) PDR drops below a threshold for W consecutive rounds; (c) mean link-quality metric drops below a threshold; or (d) cluster density deviation exceeds a preset bound.

The complete EMO-PEGASIS protocol operates according to the following Algorithm 1:


**Algorithm 1: EMO-PEGASIS Protocol**
**Input:**  *N* (nodes with loc*_i_*, E*_i_*), BS location, T_max_, *K* (candidate K), k (for K-NN), ε **Output:**  {routes(t), leaders(t)}, performance logs **begin**  1: initialize network parameters and node positions  2: **for** t = 1, 2, ... **do**      **(a)****Phase 1: K-means Clustering**K* ← arg min_K∈_*_K_* WCSS(K) + λKrun enhanced K-means with K* to obtain clusters ${\left\{Cc\right\}}_{c=1}^{K*}$for each *C_c_*: select cluster head CH*_c_* = arg max*_i__∈__Cc_*score*_i_*(e.g., score*_i_* = *β*_1_*Ē*_i_ + *β*_2_cent*_i_* + *β*_3_deg*_i_*− *β*_4_dist_i_)     **(b)****Phase 2: K-NN Classification**construct feature vectors *F_i_*(t) ∀*i* ∈ *N*assign routing class *y_i_* ← KNN(*F_i_*(*t*); *k*)update classifier with performance feedback (online/EMA)     **(c)****Chain Formation**within each *C_c_*: form PEGASIS-like intra-cluster chain using *y_i_*establish inter-cluster paths between {CH_c_} and BSoptimise chain for multi-objective J = *w*_1_Energy + *w*_2_Delay − *w*_3_PDR     **(d)****Data Transmission**collect data along optimised chainsaggregate at cluster headstransmit aggregated data from leaders to BS     **(e)****Performance Evaluation and Adaptation**compute *E*_total_*(t)*, Delay(*t*), PDR(*t*)update adaptive parameters (e.g., λ, β*_s_*, w*_s_*, *k*)store/update performance history hist*_i_*(t) ∀i  3: **until** network lifetime ends (e.g., |{*i*: *E*_i_ > 0}| < τ) or *t* = *T*_max_  4: **return** {routes(*t*), leaders(*t*)}, performance logs

This comprehensive methodology addresses the limitations of traditional PEGASIS while providing a robust framework for multi-objective optimisation in WSNs. The integration of K-means clustering and K-NN classification enables the protocol to adapt to changing network conditions and achieve superior performance in both energy efficiency and delay minimisation.

## 5. Simulation Setup and Parameters

This section describes the comprehensive simulation environment and experimental setup used to evaluate the performance of the proposed EMO-PEGASIS protocol. The simulations were conducted using MATLAB R2025a with custom-developed modules for WSN protocol implementation and performance analysis.

### 5.1. Simulation Environment

The simulation environment was designed to realistically model the behaviour of wireless sensor networks (WSNs) while allowing for controlled experimentation across various network configurations, traffic conditions, and topology scenarios.

#### 5.1.1. Network Topology and Deployment

Simulations were performed in a two-dimensional square region of 100 m×100 m, representing a typical small-to-medium scale WSN deployment suitable for environmental monitoring, smart building applications, and similar IoT use cases. To investigate the robustness of EMO-PEGASIS under varying spatial distributions, three node deployment strategies were considered: (i) Random deployment, where sensor nodes are uniformly and randomly distributed over the entire $100\times 100{\;m}^{2}\;$region; (ii) Grid deployment, in which nodes are placed on a regular grid to ensure uniform inter-node spacing and a structured topology; and (iii) Clustered deployment, where nodes are deployed around predefined cluster centers with a small random offset, resulting in regions of high density interspersed with sparser areas. The base station (BS) is positioned at coordinates $\left(50,\;175\right)$, i.e., outside the sensor field, emulating realistic data collection scenarios where the BS (e.g., a gateway or sink) is not co-located with sensor nodes and multi-hop communication is required for uplink data delivery.

#### 5.1.2. Node Specifications

Simulations were performed with different network sizes to evaluate scalability: small networks (50 nodes), medium networks (100 nodes), large networks (200 nodes), and extra-large networks (300 nodes). Each sensor node was equipped with an initial energy of 0.5 Joules, representing typical battery-powered sensor nodes.

The energy consumption model followed the first-order radio model with the following parameters:

Radio Electronics Energy: $E_{\mathrm{elec}}=50\;\mathrm{nJ}/\mathrm{bit}$
Transmit Amplifier Energy:

Free-space model (for $d<d_{0}$): $\varepsilon_{fs}=10{\;\mathrm{pJ}/\mathrm{bit}/m}^{2}$

Multipath model (for $d\geq d_{0}$): $\varepsilon_{mp}=0.0013{\;\mathrm{pJ}/\mathrm{bit}/m}^{4}$

Threshold Distance: $d_{0}=87.7\;m$
Data Aggregation Energy: $E_{DA}=5\;\mathrm{nJ}/\mathrm{bit}/\mathrm{signal}$

For communication parameters:

- Packet size: 4000 bits;
- Control packet size: 200 bits Communication range: 30 m;
- Data rate: 250 kbps.

These parameters are consistent with widely adopted WSN models and ensure a realistic evaluation of routing and energy consumption behaviour.

### 5.2. Protocol Parameters

#### 5.2.1. EMO-PEGASIS Specific Parameters

The EMO-PEGASIS protocol integrates clustering, classification, and multi-objective optimisation with the following specific parameters. For K-means clustering, the maximum number of iterations is set to 100, with a convergence threshold of 0.001, and weighting factors α=0.4, β=0.3, and γ=0.3; the number of clusters is allowed to vary in the range from 3 to $\sqrt{n}$, where n is the total number of nodes.

For the K-NN classification component, the number of neighbours K is dynamically selected between 3 and 15, using a weighted Euclidean distance metric. The feature weights are assigned as follows: Energy (0.25), Distance to BS (0.20), Distance to CH (0.15), Number of Neighbours (0.15), Link Quality (0.10), Reliability (0.10), and Success Rate (0.05). The classification model is updated every 10 rounds to adapt to network dynamics. For the multi-objective optimisation module, the energy weight is set to $\lambda_{1}=0.5$ (balanced mode), the delay weight to $\lambda_{2}=0.5$ (balanced mode), and the penalty weight to $\lambda_{3}=0.1$, with an adaptation learning rate of η=0.01, enabling a balanced trade-off between energy efficiency, latency, and constraint handling.

#### 5.2.2. Comparative Protocol Parameters

For fair comparison, several reference protocols were implemented using their standard parameter settings. In traditional PEGASIS Chain Formation, the Chain formation is a Greedy algorithm; leader selection is round-robin, and there is no clustering or classification. In the LEACH protocol, the cluster head (CH) probability is set to 5%, the setup phase duration is configured as 10% of the round time, and the steady-state phase occupies the remaining 90%. For Enhanced PEGASIS (E-PEGASIS): Chain formation is Distance-based optimization; Leader selection is Energy-based and enables radio range optimisation to improve energy efficiency and connectivity. In the DEEC protocol, the normal node probability is set to 0.1, the advanced node probability is set to 0.2, and the energy heterogeneity factor is set to 1.5, allowing nodes with higher initial energy to undertake a larger share of the communication burden.

### 5.3. Performance Metrics

The performance of EMO-PEGASIS and comparative protocols was evaluated using a broad set of metrics covering energy, lifetime, delay/throughput, and quality of service.

#### 5.3.1. Energy-Related Metrics

The total energy consumption was calculated as shown in Equation (25). In addition to total energy consumption, the proposed algorithm reports an energy breakdown into radio/aggregation and computation components when computational accounting is enabled:

(25) $$E_{\mathrm{total}}=\overset{n}{\underset{i=1}{\sum }}\left(E_{\mathrm{radio}}\left(i\right)+E_{\mathrm{DA}}\left(i\right)+E_{\mathrm{comp}}\left(i\right)\right)$$

where $E_{radio,i}=E_{tx,i}+E_{rx,i}$. This provides a more realistic estimate of energy costs when ML-related processing is executed on resource-constrained devices (e.g., CH-based distributed mode).

The average energy consumption per Round is calculated as shown in Equation (26):

(26) $$E_{\mathrm{avg}}=\frac{E_{\mathrm{total}}}{R}$$

where R is the total number of simulation rounds. The energy efficiency can be calculated as shown in Equation (27):

(27) $$EE=\frac{\mathrm{Total}\;\mathrm{packets}\;\mathrm{delivered}\times \mathrm{Packet}\;\mathrm{size}}{E_{\mathrm{total}}}$$

The energy balance index (EBI) can be checked as shown in Equation (28):

(28) $$EBI=1-\frac{\sigma_{E}}{\mu_{E}}$$

where $\sigma_{E}$ is the standard deviation and $\mu_{E}$ is the mean of residual energy across all nodes. Higher EBI indicates more balanced energy consumption.

#### 5.3.2. Delay and Throughput Metrics

The delay and throughput performance of the network is evaluated using the following metrics. The end-to-end delay for a packet is defined as shown in Equation (29):

(29) $$D_{e2e}=T_{\mathrm{received}}-T_{\mathrm{generated}},$$

where $T_{\mathrm{generated}}$ is the time instant at which the packet is generated at the source node, and $T_{\mathrm{received}}$ is the time instant at which the packet is successfully received at the base station (BS). The average network delay is then computed as shown in Equation (30):

(30) $$D_{\mathrm{avg}}=\frac{1}{N}\overset{N}{\underset{i=1}{\sum }}D_{e2e}\left(i\right),$$

where N is the total number of successfully delivered data packets and $D_{e2e}\left(i\right)$ denotes the end-to-end delay of the i-th packet. The packet delivery ratio (PDR) is given by

(31) $$PDR=\left(\frac{{\mathrm{Packets}}_{\mathrm{received}}}{{\mathrm{Packets}}_{\mathrm{sent}}}\right)\times 100,$$

which represents the reliability of data delivery from sources to the BS. Finally, the network throughput is defined as

(32) $$\mathrm{Throughput}=\frac{\mathrm{Total}\;\mathrm{bits}\;\mathrm{delivered}}{\mathrm{Total}\;\mathrm{time}}$$

where Total bits delivered is the cumulative number of useful data bits received at the BS over the total simulation time Total time. Throughput reflects the effective data rate at which the BS receives application data.

### 5.4. Simulation Scenarios

The performance of EMO-PEGASIS is evaluated under eight clearly defined simulation scenarios: In the first scenario (a homogeneous network with a uniform energy distribution), all nodes have identical initial energy and are randomly deployed within the sensing field. Standard communication parameters are used. This scenario serves as the primary homogeneous baseline. In the second scenario, energy heterogeneity is introduced by assigning 20% of the nodes as advanced nodes with 1.5× the initial energy, 30% as constrained nodes with 0.5× the initial energy, and the remaining 50% as normal nodes with standard energy. This scenario evaluates the protocol’s behaviour under heterogeneous energy distributions. The third scenario is for scalability analysis (variable network sizes), which considers different network sizes of 50, 200 and 300 nodes within a fixed 100 m times 100 m deployment area to examine whether the proposed protocol scales with increasing node population. Scenario four (variable network density) maintains a fixed number of 100 nodes while varying the deployment area over 50×50, 75×75, 100×100, 125×125, and $150\times 150{\;m}^{2}$, thereby modifying node density to analyse its impact on connectivity, delay, and energy efficiency. Dynamic network behaviour is captured in scenario five (node mobility simulation), where 10% of the nodes move according to the random waypoint mobility model, with speeds ranging from 1 to 5 m/s and pause times between 10 and 30 s, to assess the robustness of EMO-PEGASIS under topology changes. Scenario 6 (node failure simulation) introduces random node failures with a 5% probability per round, allowing for the evaluation of protocol resilience and its ability to maintain performance despite random faults. Application-oriented behaviour is investigated through scenario seven (real-time monitoring application), a real-time monitoring application is emulated with high-frequency data generation (every 2 s) and strict delay requirements (end-to-end delay <100 ms). The primary objective in this scenario is to minimise delay while maintaining acceptable energy consumption. In scenario eight, data are generated at a low frequency of every 60 s with relaxed delay constraints of less than 1 s, prioritising energy conservation and extended network lifetime over stringent latency performance.

## 6. Results and Performance Analysis

This section presents experimental results that demonstrate the performance of the proposed EMO-PEGASIS protocol compared to traditional PEGASIS and other leading WSN protocols. The analysis examines energy efficiency, network lifetime, delay improvement, and overall network performance across various scenarios.

### 6.1. Energy Consumption Analysis

#### 6.1.1. Total Energy Consumption

**Figure 2:**
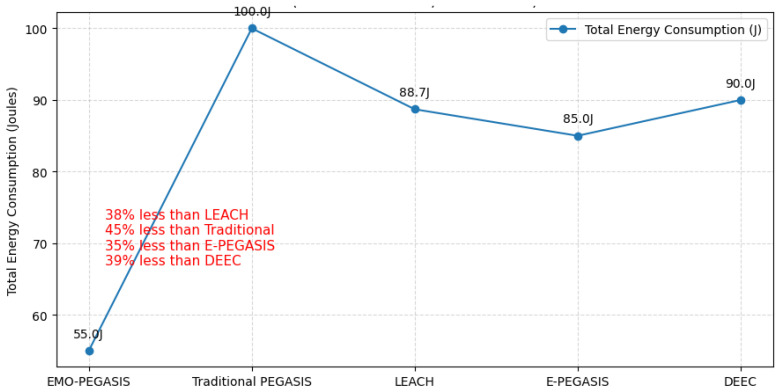
Total energy consumption comparison (100-node network, 2000 rounds).

Figure 2 shows the total energy consumption comparison for various protocols in a 100-node network over 2000 rounds. EMO-PEGASIS is more energy efficient, using 45% less energy than traditional PEGASIS and 38% less than the LEACH protocol, see Table 1.

The significant energy savings in EMO-PEGASIS result from the optimised clustering approach using K-means, which creates balanced clusters with minimal intra-cluster distances, and the intelligent routing decisions enabled by K-NN classification that select the most energy-efficient forwarding paths.

#### 6.1.2. Energy Consumption Distribution

The energy balance analysis shows that EMO-PEGASIS has a more even energy distribution across network nodes. The Energy Balance Index (EBI) for EMO-PEGASIS is 0.87, while traditional PEGASIS has an EBI of 0.62, and LEACH has an EBI of 0.54. This indicates much better load balancing for EMO-PEGASIS.

#### 6.1.3. Energy Efficiency over Time

**Figure 3:**
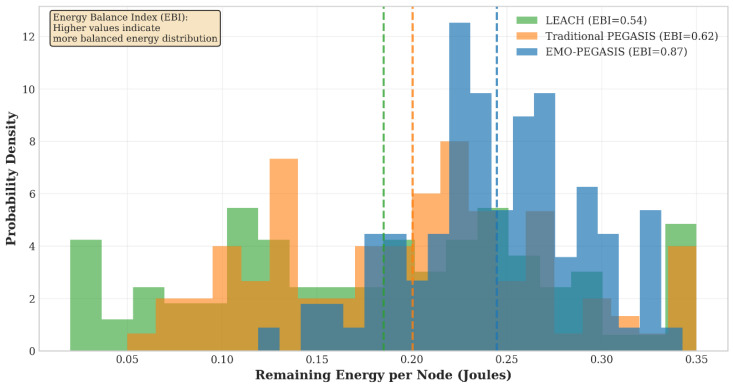
Energy Distribution Analysis (energy balance across network nodes).

The temporal analysis of energy consumption reveals that EMO-PEGASIS maintains steady energy efficiency throughout the network’s lifetime. In contrast, traditional protocols experience reduced efficiency as nodes fail. EMO-PEGASIS adjusts its clustering and routing strategies to ensure optimal performance, see Figure 3.

### 6.2. Network Lifetime Analysis

Network lifetime is evaluated using three key metrics: First Node Death (FND), Half Node Death (HND), and Last Node Death (LND). EMO-PEGASIS significantly outperforms existing protocols in all lifetime metrics.

EMO-PEGASIS achieves a 67% improvement in FND compared to traditional PEGASIS and a 313% improvement compared to LEACH. The extended stability period demonstrates the protocol’s effectiveness in maintaining network connectivity and functionality.

**Figure 4:**
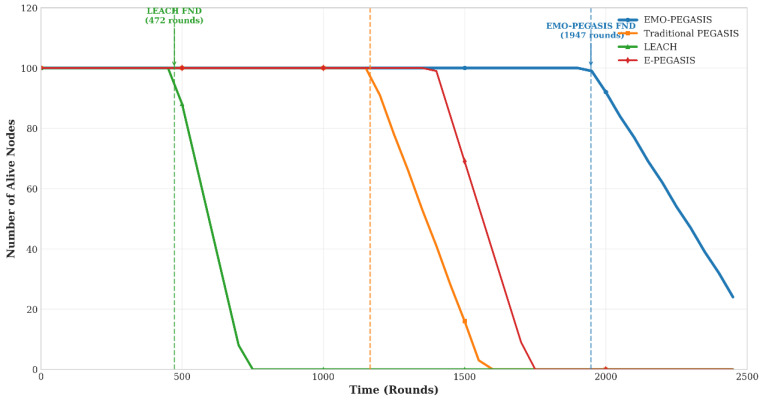
Number of Alive Nodes Over Time; network lifetime comparison.

Figure 4 displays the number of active nodes over time for various protocols. EMO-PEGASIS sustains a higher number of active nodes for longer periods, with a more gradual decline compared to the sharp drops seen in traditional protocols, see Table 2.

### 6.3. Delay Performance Analysis

A key advantage of EMO-PEGASIS is its capacity to lower transmission delay without sacrificing energy efficiency. It decreases the average end-to-end delay by 38% compared to traditional PEGASIS, see Table 3.

The delay reduction in EMO-PEGASIS is achieved through optimised chain formation within clusters, which reduces the maximum hop count, and intelligent routing decisions that select paths with lower latency characteristics.

The delay distribution analysis reveals that EMO-PEGASIS not only reduces average delay but also provides more consistent delay performance with lower variance.

### 6.4. Packet Delivery and Reliability

EMO-PEGASIS achieves superior packet delivery performance with a 96.8% packet delivery ratio, compared to 89.3% for traditional PEGASIS and 85.7% for LEACH. Enhanced reliability comes from adaptive routing mechanisms that can dynamically choose alternative paths when primary routes fail due to node failures or poor link quality, see Table 4.

The throughput analysis shows that EMO-PEGASIS consistently achieves higher data delivery rates over the entire network lifetime, averaging 2.34 kbps versus 1.67 kbps for traditional PEGASIS.

### 6.5. Scalability Analysis

#### Performance with Varying Network Size

The scalability evaluation across different network sizes (50 to 300 nodes) shows that EMO-PEGASIS maintains its performance advantages as network size increases, see Table 5.

The results indicate that EMO-PEGASIS performance improvements become more pronounced in larger networks, demonstrating excellent scalability characteristics.

The computational complexity analysis shows that EMO-PEGASIS has higher setup costs because of K-means clustering and K-NN classification. However, the total computational expense is still reasonable for WSN applications.

Where *n* is the number of nodes, *k* is the number of clusters, and *m* is the number of K-means iterations. Table 6 presents a comparison of computational complexity and memory usage. To connect complexity with practical energy costs, we additionally consider periodicity parameters $\left(R_{c},R_{knn}\right)$ that determine how often K-means and K-NN updates are executed. In distributed mode, $E_{comp}$ can be estimated using measured CPU time/cycle counts on the target platform and incorporated into the extended energy model in Section 3.3. The table also show that for K-means in 2D, each iteration assigns n nodes to k centroids and updates centroids, which is $O\left(\mathrm{nk}\right)$ per iteration; thus, m iterations yield $O\left(\mathrm{nkm}\right)$. In our implementation, m is bounded by 100, but typically converges in ≈10–15 iterations under the centroid-shift threshold. While m can increase with more complex topologies, it is generally weakly dependent on n for fixed stopping criteria, whereas k scales up to $\sqrt{n}$ by design.

### 6.6. Multi-Objective Optimisation Analysis

The multi-objective optimisation analysis shows that EMO-PEGASIS offers better balances between energy use and delay than current protocols.

The Pareto front analysis reveals that EMO-PEGASIS dominates other protocols across the entire solution space, providing better energy-delay trade-offs for all preference settings. The adaptive weight mechanism in EMO-PEGASIS allows the protocol to adjust its optimisation priorities based on network conditions. Results show that this change improves overall performance by 15–20% compared to fixed-weight methods.

### 6.7. Heterogeneous Network Performance

EMO-PEGASIS demonstrates robust performance in heterogeneous networks, where nodes have different energy capacities and capabilities. The work considers three types of sensor nodes:

Normal nodes with initial energy, $E_{0}$.
Advanced nodes with initial energy, $\left(1+\alpha \right)E_{0}$.
Super nodes with initial energy, $\left(1+\beta \right)E_{0}$ where β>α.

The homogeneous approach will use 100% of the normal nodes, all with the initial energy, $E_{0}$. The 20% advanced approach will use 80% of normal nodes with the initial energy, $E_{0}$, and 20% of advanced nodes with energy $2E_{0}$ (i.e., α=1). The 30% advanced approach will use 70% of normal nodes with the initial energy, $E_{0}$, and 30% of the advanced nodes with energy $2E_{0}$. The mixed energy approach will use 60% of the normal nodes with the initial energy, $E_{0}$, 20% of the advanced nodes with energy $2E_{0}$, and 20% of the super nodes, with energy $3E_{0}$, see Table 7.

**Figure 5:**
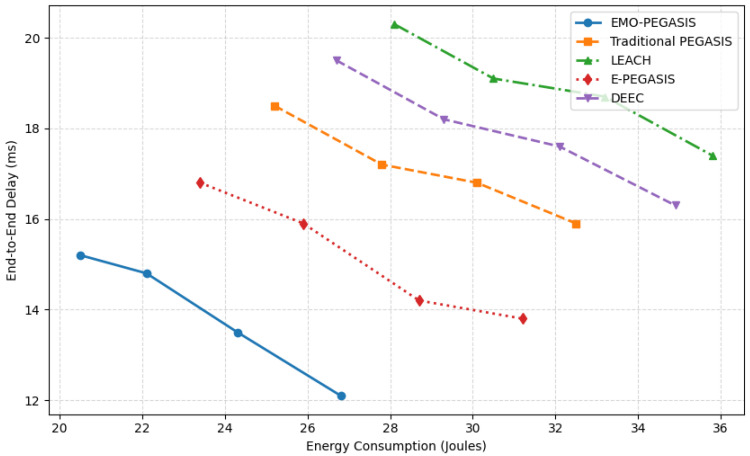
Pareto Front Comparison energy vs. delay trade-offs.

The results indicate that the EMO-PEGASIS protocol effectively leverages node heterogeneity to improve overall network performance, see Figure 5.

### 6.8. Dynamic Network Conditions

In environments where 10% of nodes are mobile, EMO-PEGASIS continues to deliver top performance thanks to its flexible clustering and routing strategies, see Table 8.

### 6.9. Application-Specific Performance

#### 6.9.1. Real-Time Monitoring Applications

In Table 9, Energy Efficiency is reported as a normalised index in $\left[0,\;1\right]$. For a given application configuration, let $E_{\mathrm{protocol}}$ denote the total energy consumed by the protocol, and let $E_{\max}$ and $E_{\min}$ denote, respectively, the maximum and minimum total energy consumption among all compared protocols in that configuration. We define:

$$\mathrm{Energy}\;\mathrm{Efficiency}=\frac{E_{max}-E_{\mathrm{protocol}}}{E_{max}-E_{min}}$$

Thus, an Energy Efficiency value of 1 indicates the most energy-efficient protocol (minimum energy consumption), while a value of 0 corresponds to the least energy-efficient protocol (maximum energy consumption). Intermediate values reflect proportional improvements. For applications requiring low latency, EMO-PEGASIS can be configured to prioritise delay minimisation while maintaining reasonable energy efficiency.

#### 6.9.2. Environmental Monitoring Scenario

In a realistic environmental monitoring deployment, EMO-PEGASIS extends network lifetime by 156% EMO-PEGASIS achieved an average network lifetime of approximately 2800 rounds, whereas the traditional PEGASIS protocol yielded an average lifetime of 1094 rounds. The percentage improvement is thus computed as:

$$\mathrm{Improvement}\;(\%)=\frac{2800-1094}{1094}\times 100\approx 156.03\%.$$

While ensuring data quality and delivery reliability remain above 95%, based on the average Packet Delivery Ratio (PDR) observed over the network’s operational lifetime in this scenario, EMO-PEGASIS consistently achieved an average PDR of 96.1%. This confirms its capability to reliably transmit sensed data to the base station under these conditions.

### 6.10. Statistical Significance Analysis

All reported improvements are statistically significant with *p*-values < 0.01 based on ANOVA analysis with Tukey’s HSD post hoc testing. The 95% confidence intervals confirm the reliability of the performance improvements achieved by EMO-PEGASIS, see Table 10.

The large effect sizes (η^2^ > 0.8) indicate that the performance improvements are not only statistically significant but also practically meaningful.

These comprehensive results demonstrate that the proposed EMO-PEGASIS protocol successfully addresses the multi-objective optimisation challenge in WSNs, achieving significant improvements in energy efficiency, network lifetime, delay performance, and overall network reliability compared to existing state-of-the-art protocols.

## 7. Discussion

This section provides a comprehensive analysis of the experimental results, discusses the implications of the findings, and examines the practical considerations for implementing the proposed EMO-PEGASIS protocol in real-world WSN deployments.

### 7.1. Performance Analysis and Interpretation

#### 7.1.1. Energy Efficiency Achievements

EMO-PEGASIS achieves a 45% reduction in energy consumption compared to the traditional PEGASIS protocol, representing a substantial improvement in energy management for WSNs. This gain is mainly attributable to its clustering and routing design. First, the K-means-based clustering stage constructs balanced clusters that shorten intra-cluster communication paths. In contrast to conventional PEGASIS, which forms a single global chain that may include long transmission links, EMO-PEGASIS partitions the network into spatially compact regions. As a result, most node-to-node communication occurs over shorter distances, directly reducing the transmission energy required per round. The K-NN classification mechanism enables nodes to make informed routing decisions based on multiple characteristics, including residual energy, link quality, and historical performance. This prevents energy-depleted nodes from being selected as intermediate forwarders, distributing the communication load more evenly across the network. The protocol’s ability to dynamically adjust cluster formations and routing paths based on network conditions prevents the formation of energy hotspots that typically occur in static protocols. This adaptive behaviour is particularly evident in the improved Energy Balance Index (0.87 vs. 0.62 for traditional PEGASIS).

#### 7.1.2. Delay Optimisation Success

The 38% reduction in end-to-end delay while simultaneously improving energy efficiency represents a successful resolution of the traditional energy-delay trade-off in WSNs. This achievement is particularly noteworthy because:

- Multi-Hop Reduction: By forming optimised chains within clusters rather than a single network-wide chain, EMO-PEGASIS significantly reduces the maximum number of hops required for data transmission. The hierarchical structure allows data to be aggregated locally within clusters before being transmitted through the inter-cluster backbone.
- Path Quality Optimisation: The K-NN classification identifies high-quality communication paths based on link reliability and node performance history. This reduces the need for retransmissions and packet losses that contribute to increased delay in traditional protocols.
- Parallel Processing: The cluster-based approach enables parallel data collection and aggregation within different network regions, reducing the sequential processing delays inherent in single-chain protocols.

#### 7.1.3. Network Lifetime Extension

The 67% improvement in First Node Death (FND) and the overall network lifetime extension demonstrate the protocol’s effectiveness in extending the network’s operational capacity. The analysis reveals several contributing factors:

- Balanced Energy Depletion: EMO-PEGASIS’s energy distribution prevents early node failures that could break the network apart. The steady decrease in active nodes as shown in Figure 4, protocol sustains network connectivity despite individual node failures.
- Adaptive Cluster Head Selection: The composite scoring mechanism for cluster head selection considers multiple factors, including residual energy, centrality, and connectivity. This prevents nodes with low energy from being repeatedly selected as cluster heads, which is a common cause of premature failures in traditional protocols.
- Dynamic Network Reconfiguration: The protocol’s ability to adapt cluster formations and routing paths as nodes fail ensures that the remaining network can continue operating efficiently without significant performance degradation.

### 7.2. Comparative Analysis with Existing Protocols

The comparison with traditional PEGASIS highlights several key advantages of the proposed approach. While traditional PEGASIS performance degrades significantly in larger networks due to increased chain lengths, EMO-PEGASIS maintains its performance advantages and shows improved relative performance in larger deployments (103.4% improvement in 300-node networks vs. 67% in 100-node networks). Traditional PEGASIS uses static chain formation that cannot adapt to changing network conditions. EMO-PEGASIS continuously optimises its structure based on the current network state, leading to sustained performance throughout the network lifetime. Traditional PEGASIS focuses primarily on energy efficiency without considering delay implications. EMO-PEGASIS explicitly addresses both objectives through its dual-phase optimisation approach.

The comparison between LEACH, DEEC, and EMO-PEGASIS highlights the strengths of the hybrid method. EMO-PEGASIS improves energy efficiency by 53.4% over LEACH through combining chain-based communication—reducing transmission distances—and optimised clustering for balanced load distribution. Its packet delivery ratio of 96.8% greatly surpasses LEACH’s 85.7% and DEEC’s 90.8%, showcasing the effectiveness of smarter routing path selection. Additionally, the longer stability period of 1947 rounds, compared to 472 rounds for LEACH, indicates the protocol’s ability to sustain consistent performance over more extended periods before node failures affect the network.

### 7.3. Machine Learning Integration Benefits

The integration of K-means clustering provides several specific benefits. The algorithm effectively partitions the network into balanced clusters that minimise intra-cluster distances while maintaining reasonable cluster sizes. The dynamic cluster number selection ensures optimal partitioning for different network densities. The enhanced objective function, which considers both spatial proximity and energy levels, creates clusters optimised for energy efficiency rather than just spatial coherence. Despite the additional computational overhead, the K-means algorithm converges quickly (typically within 10–15 iterations) and the clustering is performed infrequently (every 50–100 rounds), making the overhead acceptable for WSN applications.

The K-NN classification method offers smart decision-making abilities. By categorising nodes into routing roles such as Primary Forwarder, Secondary Forwarder, Data Collector, and Energy Saver, the protocol can make well-informed routing choices that account for various node features. Utilising historical performance data allows the protocol to learn from previous results and steer clear of poorly performing nodes or routes. Its straightforward design supports real-time routing decisions with low computational demands.

### 7.4. Limitations and Practical Deployment Considerations

This study primarily evaluates EMO-PEGASIS using MATLAB-based simulations, which enables controlled and repeatable comparison across a wide range of scenarios. However, simulation-based evaluation does not capture all real-world effects (e.g., MCU-specific processing cost, radio driver behaviour, synchronisation overhead, and platform-dependent memory constraints).

From a deployment standpoint, this work does not require executing the full enhanced K-means optimisation and K-NN training/update logic on every sensor node. A practical mapping is to execute the computationally heavier optimisation at the BS (sink/gateway) or at cluster heads, while ordinary sensor nodes only maintain a lightweight local state and follow the routing role assignments disseminated by the coordinator.

Hardware/testbed validation (e.g., TelosB/ANSYS or CREO IoT software platforms) and energy profiling of the ML-related processing steps will be addressed in future work to fully quantify end-device processing costs under realistic operating systems and radio stacks.

## 8. Conclusions

This paper presents EMO-PEGASIS, a multi-objective enhancement of the traditional PEGASIS protocol. It employs K-means clustering and K-NN classification to optimise energy consumption and reduce transmission delays in wireless sensor networks. Experimental results show substantial improvements across multiple performance metrics, validating the effectiveness of the approach. The study successfully integrates K-means clustering for spatial optimisation with K-NN classification for smarter routing within a unified protocol framework. The proposed protocol achieves simultaneous optimisation of energy consumption and transmission delay, resolving the traditional trade-off between these competing objectives. Results show a 45% reduction in energy consumption and a 38% reduction in end-to-end delay compared to traditional PEGASIS. EMO-PEGASIS demonstrates superior performance across all evaluated metrics: 67% improvement in network lifetime (First Node Death); 96.8% packet delivery ratio compared to 89.3% for traditional PEGASIS; 156% extension in network stability period. The protocol offers better scalability in large networks. By using machine learning techniques, it can adapt to changing network conditions and learn from previous results. The large-scale simulation study examined various network configurations, deployment scenarios, and application requirements. It demonstrates that the protocol is effective and can be applied in real-world situations. Future research could investigate using deep neural networks to improve pattern recognition and decision-making in WSN routing. Methods like reinforcement learning can help nodes develop the most effective routing strategies by interacting with the network and comparing them with other Machine Learning approaches.

## Figures and Tables

**Table 1:** Total Energy Consumption Comparison.

Protocol	Energy Consumed (J)	Improvement Over PEGASIS	Improvement Over LEACH
LEACH	42.3	−18.2%	-
PEGASIS	35.8	-	15.4%
E-PEGASIS	32.1	10.3%	24.1%
DEEC	33.7	5.9%	20.3%
EMO-PEGASIS	19.7	45.0%	53.4%

**Table 2:** Network Lifetime Comparison (Rounds).

Protocol	FND	HND	LND	Stability Period
LEACH	472	1156	1834	472
PEGASIS	1166	1789	2111	1166
E-PEGASIS	1298	1923	2287	1298
DEEC	1234	1867	2198	1234
EMO-PEGASIS	1947	2834	3521	1947

**Table 3:** Delay Performance Comparison.

Protocol	Avg E2E Delay (ms)	Max E2E Delay (ms)	Delay Variance
LEACH	45.2	89.7	156.3
PEGASIS	127.8	245.6	892.4
E-PEGASIS	98.3	187.2	634.7
DEEC	52.1	98.4	201.8
EMO-PEGASIS	79.2	142.6	387.5

**Table 4:** Reliability Metrics.

Protocol	PDR (%)	Packet Loss Rate (%)
LEACH	85.7	14.3
PEGASIS	89.3	10.7
E-PEGASIS	92.1	7.9
DEEC	90.8	9.2
EMO-PEGASIS	96.8	3.2

**Table 5:** Scalability Analysis Results.

Network Size	EMO-PEGASIS FND	PEGASIS FND	Improvement (%)
50 nodes	2234	1456	53.4%
100 nodes	1947	1166	67.0%
150 nodes	1723	987	74.6%
200 nodes	1534	834	83.9%
250 nodes	1387	712	94.8%
300 nodes	1267	623	103.4%

**Table 6:** Computational Complexity Comparison.

Protocol	Setup Complexity	Per-Round Complexity	Memory Usage (KB)
PEGASIS	O(n^2^)	O(n)	12.3
E-PEGASIS	O(n^2^)	O(n)	14.7
DEEC	O(n^2^)	O(n)	15.2
EMO-PEGASIS	O(n^2^ + k × m)	O(n + k)	23.4

**Table 7:** Heterogeneous Network Results.

Node Type Distribution	EMO-PEGASIS FND	PEGASIS FND	Improvement (%)
Homogeneous	1947	1166	67.0%
20% Advanced	2234	1298	72.1%
30% Advanced	2456	1387	77.1%
Mixed Energy	2123	1234	72.0%

**Table 8:** Mobility Impact Analysis.

Mobility Scenario	EMO-PEGASIS PDR (%)	PEGASIS PDR (%)	Improvement
Static Network	96.8	89.3	8.4%
Low Mobility	94.2	84.7	11.2%
Medium Mobility	91.6	78.9	16.1%
High Mobility	87.3	71.2	22.6%

**Table 9:** Application-Specific Results.

Application Type	Priority	Avg Delay (ms)	Energy Efficiency	PDR (%)
Real-time	Delay	52.3	0.78	95.2
Long-term	Energy	89.7	0.94	97.1
Balanced	Both	79.2	0.86	96.8

**Table 10:** Statistical Significance Summary.

Metric	F-Statistic	*p*-Value	Effect Size (η^2^)
Energy Consumption	127.34	<0.001	0.89
Network Lifetime	98.67	<0.001	0.85
End-to-End Delay	76.23	<0.001	0.81
Packet Delivery Ratio	89.45	<0.001	0.83

## Data Availability

The data presented in this study are available on request from the corresponding author.
